# 1024. Real-world Adherence and Persistence with Long-Acting Cabotegravir Plus Rilpivirine (CAB+RPV LA) Compared to Oral Antiretroviral Therapy (ART) Among People with HIV (PWH) in the US: The ABOVE Study

**DOI:** 10.1093/ofid/ofad500.055

**Published:** 2023-11-27

**Authors:** Cindy Garris, Raj Desai, Rose Chang, Louise Clear, Zhuo Chen, Daisy Liu, Maral DerSarkissian, Paula Teichner, Edgar T Overton

**Affiliations:** ViiV Healthcare, Durham, NC; Analysis Group, Inc., Boston, Massachusetts; Analysis Group, Inc., Boston, Massachusetts; Analysis Group, Inc., Boston, Massachusetts; Analysis Group, Inc., Boston, Massachusetts; Analysis Group, Inc., Boston, Massachusetts; Analysis Group, Inc., Boston, Massachusetts; ViiV Healthcare, Durham, NC; ViiV Healthcare, Durham, NC

## Abstract

**Background:**

CAB+RPV LA is the only complete long-acting regimen for treatment of virologically suppressed PWH. Administered monthly or every 2 months by a healthcare provider, CAB+RPV LA may alleviate adherence challenges with daily oral therapy. The ABOVE study evaluated real-world adherence and persistence to CAB+RPV LA versus remaining on oral ART regimens.

**Methods:**

ABOVE was a retrospective US cohort study using Symphony Health Solutions Integrated Dataverse administrative claims database from 01/01/2020 to 12/31/2022. PWH ≥12 years of age on stable guideline-recommended oral ART were categorized into those initiating CAB+RPV LA and those remaining on oral ART. Index date was defined as first injection between 01/01/2021 and 6/30/2022 (CAB+RPV LA cohort) or imputed for the oral ART cohort. PWH were required to have ≥6 months of follow-up after index. Standardized mortality ratio (SMR) weights were generated based on propensity scores to balance baseline characteristics between cohorts. Adherence (proportion of days covered (PDC) ≥0.9 over 6-months following index) and persistence to the index regimen (days from index to the earliest of treatment discontinuation or end of follow-up) were compared. Logistic regression model was used to estimate the odds ratio (OR) and 95% confidence interval (CI) for adherence.

**Results:**

393,484 PWH were identified during the study period. After applying eligibility criteria, 130,362 in the oral ART cohort (N=950 after weighting) and 947 in the CAB+RPV LA cohort comprised the analysis sample. Key baseline characteristics were balanced post SMR weighting (Table 1). Majority of CAB+RPV LA dosing was every two months only (50%) or switched from monthly to every two months (33%). A higher proportion of PWH in the CAB+RPV LA cohort were adherent (72% vs 43%, p< 0.001) and had higher persistence (274 vs 256 days, p< 0.001) compared with the oral ART cohort (Table 2). PWH in the CAB+RPV LA cohort had significantly higher adjusted odds of being adherent compared to the oral ART cohort (OR: 4.43, 95% CI: 2.38, 8.24, p< 0.001).

**Figure:**
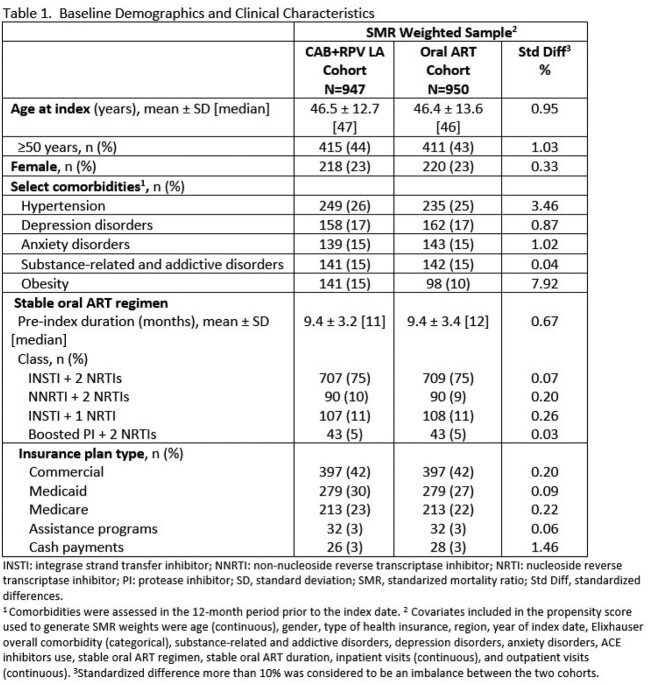

**Figure:**
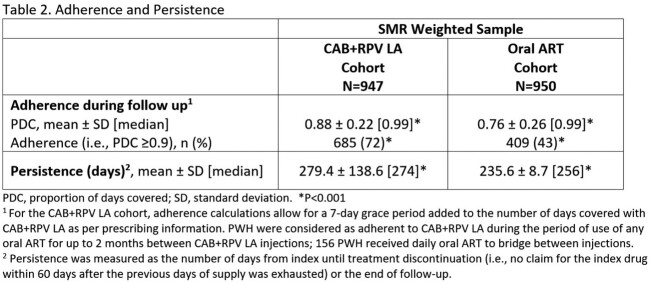

**Conclusion:**

These data demonstrate that, among US PWH on stable oral ART, switching to long-acting ART resulted in significantly higher adherence and persistence, a critical aspect of long-term success of HIV treatment, compared with remaining on oral ART.

**Disclosures:**

**Cindy Garris, MS**, GSK: Stocks/Bonds|ViiV Healthcare: Employee **Raj Desai, PhD**, Analysis Group, Inc: Employment|ViiV Healthcare: Grant/Research Support **Rose Chang, ScD**, Analysis Group, Inc.: Employee|ViiV Healthcare: Grant/Research Support **Louise Clear, MPH**, Analysis Group, Inc.: Employment|ViiV Healthcare: Grant/Research Support **Zhuo Chen, MPH**, Analysis Group, Inc.: Employment|ViiV Healthcare: Grant/Research Support **Daisy Liu, MS**, Analysis Group, Inc.: Employment|ViiV Healthcare: Grant/Research Support **Maral DerSarkissian, PhD**, Analysis Group, Inc: Employment|ViiV Healthcare: Grant/Research Support **Paula Teichner, PharmD**, GlaxoSmithKline: Stocks/Bonds|ViiV Healthcare: Employment **Edgar T. Overton, MD**, ViiV Healthcare: Employment|ViiV Healthcare: Stocks/Bonds

